# Targeting carbonic anhydrase IX-mediated ferroptosis by atractylenolide I alleviates DSS-induced colitis in mice

**DOI:** 10.3389/fphar.2026.1860254

**Published:** 2026-07-27

**Authors:** Yan Wang, Min Liang, Hanqing Guo, Zhenxi Sun, Cheng Sun, Mingxian Chen, Luzhou Xu

**Affiliations:** 1 Department of Gastroenterology, Affiliated Hospital of Nanjing University of Chinese Medicine (Jiangsu Province Hospital of Chinese Medicine), Nanjing, Jiangsu, China; 2 The Integrated Traditional Chinese and Western Medicine School of Clinical Medicine, Zhejiang Chinese Medical University, Hangzhou, Zhejiang, China

**Keywords:** Atractylenolide I, carbonic anhydrase IX, DSS-induced colitis, ferroptosis, inflammatory bowel disease

## Abstract

**Background:**

While the involvement of ferroptosis in the pathological progression of chronic inflammatory bowel disease (IBD) is recognized, the specific regulatory capacity of the natural derivative Atractylenolide I (ATT-I) within this metabolic framework is not yet fully elucidated.

**Objective:**

To investigate how ATT-I alleviates IBD by modulating ferroptosis via targeting carbonic anhydrase IX (CA9).

**Methods:**

To determine the therapeutic potential of ATT-I, a murine model of colitis was established via dextran sulfate sodium (DSS) administration. Mice were treated with different doses of ATT-I. At the end of the experiment, colon tissues and serum were collected for histological analysis, inflammatory cytokine measurement, and ferroptosis marker evaluation. Erastin (ferroptosis inducer) and adenovirus associated virus 9 (AAV9)-mediated CA9 silencing was applied to assess its role in ATT-I efficacy.

**Results:**

Medium and high doses of ATT-I significantly alleviated DSS-induced colitis symptoms by reducing histological damage, preventing colon shortening, decreasing spleen index and cytokine levels, and improving epithelial integrity. ATT-I inhibited ferroptosis by upregulating GPX4 and SLC7A11 and downregulating COX-2 and ACSL4. Notably, co-administration of Erastin reversed the protective effects of ATT-I. Network pharmacology and molecular docking suggested CA9 as a putative binding target of ATT-I which was confirmed by CETSA demonstrating increased thermal stability of CA9 upon ATT-I treatment. To investigate the functional necessity of CA9, AAV9 was employed to silence its expression. In a murine model of DSS-induced colitis, the therapeutic benefits of ATT-I, specifically its capacity to prevent splenic hypertrophy, alleviate leukocyte infiltration, and preserve colonic morphology, were entirely abolished following the genetic knockdown of CA9. Furthermore, the capacity of ATT-I to inhibit ferroptosis in the colon was obviated when CA9 was silenced. For the *in vitro* experiments, silencing CA9 abrogated ATT-I-mediated Claudin-1, Occludin, GPX4 and SLC7A11 upregulation in Erastin-treated Caco-2 cells, indicating that CA9 is an indispensable mediator for ATT-I to restrain Erastin-induced ferroptosis in Caco-2 cells.

**Conclusion:**

ATT-I alleviates IBD by modulating ferroptosis mechanisms through targeting CA9. This indicates that ATT-I, as a natural compound, holds potential for regulating ferroptosis, providing a novel therapeutic strategy and approach for IBD treatment.

## 1 Introduction

Characterized by cyclical inflammatory manifestations throughout distinct regions of the gastrointestinal tract, the clinical spectrum of inflammatory bowel disease (IBD) encompasses ulcerative colitis (UC) and Crohn’s disease (CD). This pathological condition represents a major international medical challenge, defined by persistent intestinal inflammation and a documented escalation in worldwide epidemiological frequency (M'Koma, 2013). This chronic inflammation poses a substantial risk for the development of colorectal carcinoma (Kotla and Rochev, 2023). Notwithstanding contemporary medical progress, effective treatment modalities for IBD remain limited, necessitating the urgent formulation of innovative and more potent therapeutic strategies to enhance patient prognosis. Ferroptosis, distinguished from conventional regulatory cell death like necrosis or apoptosis, is driven by the fatal accumulation of iron-mediated lipid peroxidation and extensive oxidative damage (Nirmala and Lopus, 2020). As a distinct programmed cell death mechanism, it has emerged as a focal point of recent research, offering new insights beyond classical pathways. In the context of IBD, chronic inflammation serves as a primary driver of excessive intestinal epithelial cell death and subsequent barrier impairment (Söderholm et al., 2002). Specifically, ferroptosis acts as a critical mediator of this epithelial cell death, thereby exacerbating barrier dysfunction (Chen et al., 2021). Research involving IBD patients has highlighted specific ferroptotic signatures, including disrupted iron homeostasis, depleted glutathione levels, and diminished GPX4 activity, which ultimately trigger an explosion of lipid reactive oxygen species (ROS) (Gao et al., 2021; Lei et al., 2021). Consequently, suppressing the ferroptotic process through genetic or chemical means can effectively alleviate colitis and promote the healing of the gut lining, underscoring its potential as a therapeutic target (Huang et al., 2022; Xu et al., 2021; Zhang et al., 2023).

Derived from the roots of *Atractylodes macrocephala*, Atractylenolide I (ATT-I) serves as a major bioactive sesquiterpene lactone. Extensive research has validated its multifaceted pharmacological profile, encompassing anti-oxidative, anti-inflammatory, and tumor-suppressive properties, which underscores its potential across diverse disease models (Qin et al., 2021; Li et al., 2020; Wang et al., 2020). Beyond its known neuroprotective effects, such as curbing apoptosis in human neuroblastoma cells (More and Choi, 2017), and its systemic anti-inflammatory actions (Du et al., 2022; Wen and Xiao, 2024), ATT-I has been shown to preserve intestinal homeostasis by countering antibiotic-driven microbial dysbiosis (Liu et al., 2021). Despite these advancements, whether ATT-I can modulate ferroptosis to alleviateIBD remains to be elucidated.

The present research aims to clarify how ATT-I exerts its therapeutic effects on inflammatory bowel disease (IBD) via ferroptosis regulation. Our findings pinpoint carbonic anhydrase IX (CA9) as a primary molecular interactor of ATT-I. While carbonic anhydrases generally facilitate the interconversion of CO_2_ and HCO_3_^−^, CA9 is a membrane-bound isoenzyme traditionally associated with pH homeostasis in tumor niches (McDonald et al., 2019; Van Kuijk et al., 2016; Giatromanolaki et al., 2020). Utilizing a combination of molecular docking, cellular thermal shift assays (CETSA), and AAV-mediated knockdown, we confirmed that ATT-I anchors to CA9. This interaction appears to drive the compound’s potent anti-inflammatory and anti-ferroptotic actions, ultimately reversing IBD pathology. Thus, targeting the ATT-I/CA9 axis represents a promising pharmacological avenue for IBD intervention.

## 2 Materials and methods

### 2.1 Animals

Procured from Charles River (Beijing, China), C57BL/6 male mice (18–22 g body weight) at 6–8 weeks of age were utilized for this study. Environmental maintenance occurred within a specific pathogen-free (SPF) facility, where animals were provided unrestricted availability of standard diet and hydration under a synchronized 12-h photoperiod. All procedural interventions received formal authorization from the Nanjing University of Chinese Medicine Animal Use Committee (No. 202509A109). For euthanasia and terminal sampling, systemic anesthesia was achieved through the administration of an intraperitoneal cocktail comprising acetylpromazine (2.5 mg/kg) and ketamine (100 mg/kg). The depth of sedation was ascertained by the disappearance of the pedal withdrawal reflex. Subsequently, intracardiac blood was harvested from mice in the supine position by performing a vertical sternal puncture with a 22-gauge needle. The maximum possible volume of blood was withdrawn. This procedure is typically terminal. If any animal remained alive after blood collection, euthanasia was immediately performed by cervical dislocation.

### 2.2 Dextran sulfate sodium (DSS)-Induced colitis and treatments

The animal experiments were carried out in compliance with the ARRIVE guidelines 2.0 to assure the adequate management of animals (Percie du Sert et al., 2020). An animal model of colitis was induced by providing mice with 3% dextran sulfate sodium (DSS, M.W 40,000, Sigma Chemical Co., United States) in their drinking water for a 7-day duration. Based on a previous methodology (Zhang et al., 2020), animals were randomized into six experimental cohorts (n = 8 each): a healthy Control, a DSS-only Model, a positive control group receiving Sulfasalazine (SASP, 200 mg/kg via gavage), and three ATT-I (Macklin, A860603, China) intervention groups administered intraperitoneally at dosages of 10, 20, and 40 mg/kg (saline containing 0.1% DMSO) from day 0 to day 10 once every day. A ferroptosis inducer Erastin was used to evaluate the effect of ATT-I on ferroptotic damage in DSS-induced colitis. Mice were randomized into four experimental cohorts (n = 6): Normal, DSS-only, DSS + ATT-I (40 mg/kg, i. p.), DSS + ATT-I (40 mg/kg, i. p.) + Erastin (40 mg/kg, i. p., Macklin, E872563) according to the previous study (Deng et al., 2025). Clinical progression was monitored through daily recordings of body mass and the disease activity index (DAI). The DAI, a composite metric for assessing colitic severity, integrated scores for relative weight fluctuations, stool texture, and occult blood presence (Richeldi et al., 2017). Specifically, weight loss was scored from 0 to 4 (0: stable; 1: 5%–10%; 2: 11%–15%; 3: 16%–20%; 4: >20%), while stool consistency and hematochezia were graded as follows: normal (0), loose (2), or diarrhea (4) for the former, and absence (0) or presence (4) for the latter. To facilitate post-experimental analyses, mice were anesthetized via an intraperitoneal administration of 5% sodium thiopental prior to sacrifice. Following systemic blood collection, the colonic length and splenic mass were accurately determined. For microscopic assessment, 5-mm mid-sections of the colonic tissue were excised and briefly irrigated with phosphate-buffered saline (pH 7.4). These specimens were then transitioned into a 10% formalin solution to ensure optimal fixation for subsequent histopathological examination.

### 2.3 Histological analysis

Paraffin-embedded blocks were prepared following the standard dehydration and 24-h fixation of excised colonic specimens in 10% neutral buffered formalin. From these processed tissues, transverse sections with a thickness of 5 µm were generated and subjected to hematoxylin and eosin (H&E) histological staining (G1120, Solarbio Life Sciences, China). Microscopic analysis was employed to assess histopathological changes, with damage graded according to a previously established system (Sang et al., 2015). The degree of histological injury was quantified on a 0–3 scale using the following parameters: no damage (0), localized epithelial focal lesions (1), mucosal erosions/ulcerations (2), and extensive transmural impairment (3). Simultaneously, leukocytic infiltration in the lamina propria was graded from 0 to 3: rare (0), elevated presence including neutrophils (1), submucosal clusters (2), and transmural infiltration (3). The composite histopathological score was derived by summing these subscores (total range: 0–6). For Alcian Blue periodic acid Schiff (AB-PAS) staining, 5 µm thickness colon section was covered with Alcian Blue staining solution for 10 min. After washing three times with distilled water the sections were immersed in PAS oxidant for 5 min. Schiff staining solution was applied to cover the tissue for 10 min, followed by hematoxylin staining solution for 2 min. Finally, Scott’s bluing solution was used for 3 min to turn nuclei blue (G1285, Solarbio Life Sciences, China). Microscopic analysis was employed to assess number of goblet cells.

### 2.4 FITC-dextran permeability assay

At the last day of experiments, the fluorescein isothiocyanate (FITC)-labeled dextran solution was prepared using PBS. Each group of mice was then administered 750 mg/kg of FITC-dextran (CA 1810, Solarbio Life Sciences, China) via oral gavage (Li et al., 2023). Serum FITC-dextran concentrations were determined spectrophotometrically after a 4-h period, with fluorescence monitored at excitation/emission peaks of 485/528 nm. The intensity data were subsequently translated into molar concentrations by referencing a pre-calibrated standard curve.

### 2.5 Diamine oxidase (DAO) activity assay

Quantification of diamine oxidase (DAO) activity was performed using a Bio-Tek microplate reader to detect the optical density (OD) at a wavelength of 450 nm following the enzymatic reaction. The experimental procedure utilized specialized ELISA kits sourced from Solarbio (BC1285, Beijing, China), with all analytical steps executed in rigorous accordance with the manufacturer’s standardized protocols. The resultant absorbance data were then interpolated from a pre-established standard curve to determine the absolute DAO concentrations for each sample.

### 2.6 Quantitative PCR (qPCR) analysis

To quantify the transcript levels of various inflammatory cytokines (TNF-α, IFN-γ, IL-1β, IL-18, IL-6, and IL-23), colonic mRNA expression was analyzed through quantitative PCR. The process initiated with the isolation of total colonic RNA utilizing TRIzol reagent (Thermo Fisher Scientific, United States) in strict accordance with the manufacturer’s protocols. Following the assessment of RNA quality and quantity via NanoDrop spectrophotometry, a commercial kit was employed for reverse transcription into cDNA (Cat.No. CW2569M, CWBIO, China). Real-time amplification was performed using SYBR Green Master Mix (Vazyme Biotech, China), with 2^−ΔΔCT^ calculations utilized to determine relative fold-changes. qPCR was performed with an initial denaturation at 95 °C for 30 s, 40 amplification cycles (95 °C for 10 s, 65 °C for 30 s), followed by melting curve acquisition (95 °C for 15 s, 65 °C for 60 s, 97 °C for 1 s) and cooling at 37 °C for 30 s. GAPDH served as the internal normalization control, and detailed sequences for all primers are documented in Table 1.

**TABLE 1:** Primer sequence.

Gene	Forward (5′-3′)	Reverse (5′-3′)
GAPDH	GGA​GAG​TGT​TTC​CTC​GTC​CC	ATG​AAG​GGG​TCG​TTG​ATG​GC
TNF-α	CCC​TCA​CAC​TCA​CAA​ACC​AC	ACA​AGG​TAC​AAC​CCA​TCG​GC
IFN-γ	GAG​GTC​AAC​AAC​CCA​CAG​GT	GGG​ACA​ATC​TCT​TCC​CCA​CC
IL-1β	TGC​CAC​CTT​TTG​ACA​GTG​ATG	ATG​TGC​TGC​TGC​GAG​ATT​TG
IL-18	CCC​TTT​GAG​GCA​TCC​AGG​AC	TGG​GAA​CAG​CCA​GTG​TTC​AG
IL-6	GAC​AAA​GCC​AGA​GTC​CTT​CAG​A	TGT​GAC​TCC​AGC​TTA​TCT​CTT​GG
IL-23	CAA​AGG​ATC​CGC​CAA​GGT​CT	GGA​GGT​GTG​AAG​TTG​CTC​CA

### 2.7 Western blot analysis

To obtain total protein lysates, excised colonic tissues were homogenized in RIPA buffer (Beyotime, China) supplemented with protease and phosphatase inhibitors. Quantification of the resulting protein was performed via a BCA assay (Solarbio). Subsequently, standardized amounts of protein were separated according to molecular weight through SDS-PAGE before being transferred onto PVDF membranes. These membranes underwent a 1-h incubation in a 5% skim milk blocking solution, followed by an overnight primary antibody challenge at 4 °C using the following specific targets: Claudin-1 (ab317709), Occludin (ab216327), COX-2 (ab283574), and GPX4 (ab125066), all sourced from Abcam; TNF-α (17590-1-AP), IL-1β (26048-1-AP), Carbonic anhydrase IX (11071-1-AP) SLC7A11 (26864-1-AP), β-actin (66009-1-Ig), β-tubulin (10094-1-AP) from Proteintech (China); and ACSL4 (#38493) and SLC7A11 (#98051) from Cell Signaling Technology (CST). Relative protein quantification was performed by densitometric analysis of bands using ImageJ software, with molecular weights determined by a Bio-platform (Shanghai, China) protein ladder. Prior to this, the Bio-Rad Chemidoc MP System was utilized for chemiluminescent signal acquisition following a secondary incubation step involving HRP-conjugated antibodies.

### 2.8 ELISA

Pro-inflammatory cytokine profiling was performed by homogenizing colonic tissues in chilled phosphate-buffered saline (PBS), with the resulting supernatants harvested via centrifugation. Quantification of the resulting protein was performed via a BCA assay (Solarbio). The protein abundance of TNF-α (Cat.No. CSB-E04741m), IFN-γ (Cat.No. CSB-E04578m-IS), IL-1β (Cat.No. CSB-E08054m), IL-18 (Cat.No. CSB-E04609m), IL-6 (Cat.No. CSB-E04639m), and IL-23 (Cat.No. CSB-E08463m) was subsequently quantified utilizing commercial ELISA kits (CUSABIO, China) according to the supplier’s established protocols. Quantification of specific concentrations within each specimen was achieved through interpolation from a pre-established standard curve. This analytical process involved the measurement of resultant absorbance data at a 450 nm wavelength via a microplate reader.

### 2.9 Detection of ferroptosis-related markers

Quantification of ferrous iron (Fe^2+^, BC5415, Solarbio) and malondialdehyde (MDA, BC6415, Solarbio) levels, along with the assessment of glutathione (GSH, BC1175, Solarbio) and superoxide dismutase (SOD, BC6415, Solarbio) enzymatic activities, was conducted using colonic tissue supernatants. These samples were prepared by homogenizing the specimens followed by a 10-min centrifugation cycle at 4 °C and 5000×g. All analytical procedures were performed in rigorous accordance with the standardized manuals accompanying the specific commercial biochemical assay kits.

### 2.10 TUNEL assay

*In situ* DNA fragmentation was scrutinized to assess colonic apoptosis utilizing a commercial TUNEL detection kit (Roche, Germany) in strict accordance with the supplier’s established protocols. Briefly, deparaffinized and rehydrated colonic tissue sections were treated with proteinase K. TUNEL was performed on colonic tissue slices by applying the specific reaction reagents containing terminal deoxynucleotidyl transferase and fluorescein-labeled dUTP for a 1-h incubation period at 37 °C. The nuclei were counterstained with DAPI for 5 min at room temperature. A fluorescence microscope was then employed to detect and capture images of the apoptotic nuclei.

### 2.11 Confocal laser scanning microscopy

For immunofluorescence analysis, colonic tissue sections (5 μm thickness) were deparaffinized, rehydrated, and subjected to antigen retrieval by heating in sodium citrate buffer for 10 min. The sections were then blocked with 5% bovine serum albumin (BSA) in PBS for 1 h at room temperature. Subsequently, the sections were incubated overnight at 4 °C with anti-EpCAM antibody (Proteintech, 21050-1-AP, 1:200) and anti-GPX4 antibody (Proteintech, 67763-1-Ig, 1:400). After washing three times, the sections were incubated with fluorescence-conjugated secondary antibodies (Alexa Fluor 488-conjugated anti-rabbit and Alexa Fluor 594-conjugated anti-mouse, 1:200) for 1 h at room temperature in the dark. Nuclei were counterstained with DAPI for 3 min. Finally, the sections were examined under a confocal laser scanning microscope (3DHISTECH, PANNORAMIC MIDI).

### 2.12 Molecular docking

Potential ATT-I targets were first predicted via structure-based virtual screening using the Swiss Target Prediction tool. These candidates were further refined by cross-referencing with IBD- and ferroptosis-associated genes sourced from the Genecards database. Intersecting targets were subsequently isolated through Venn analysis. Finally, binding affinities between ATT-I and the identified proteins were evaluated via molecular docking simulations using AutoDock software.

### 2.13 Cell culture and treatment

Human Caco-2 cell line was purchased from National Collection of Authenticated Cell Cultures (China) and cultured in Minimum Essential Medium (MEM) supplemented with 10% FBS, 100 U/mL penicillin and 100 μg/mL streptomycin (Gibco, United States) at 37 °C under 5% (v/v) CO_2_ atmosphere. Caco-2 cells were subjected to 20 μM Erastin treatment to induce a ferroptosis condition.

### 2.14 Cell counting kit-8 (CCK-8) assay

Cells were seeded at a density of 10^3^ per well (96-well plate) and subjected to specific stimulation conditions as indicated. After culturing for 24 h, 10 μl CCK-8 solution (CA1212, Solarbio Life Sciences, China) was added into each well and incubated for 1 h at 37 °C. The absorbance at 450 nm wavelength was detected by a microplate reader (Thermo Fisher Scientific, United States).

### 2.15 Cell transient transfection

Transfection with small interfering RNA (siRNA) was performed using FuGENE® SI Transfection Reagent (E9311, Promega, United States) according to the manufacturer’s instructions. Cells were seeded at a density of 2 × 105 per well (6-well plate). The transfection complex was prepared by diluting 3.0 µl of FuGENE® SI reagent in 47 µl of serum-free transfection medium, followed by the addition of 25 pmol of siRNA (diluted in 50 µl of serum-free medium). The mixture was incubated at room temperature for 5 min and added to the cells. At 24 h post-transfection, the culture medium was replaced with fresh medium for further treatment. Synthetic siRNA targeting CA9 (sense: 5′- CAGCCGCTACTTC CAATAT-3’; antisense: 5′ATA​TTG​GAA​GTA​GCG​GCT​G-‘) was obtained from GenePharma (Shanghai, China). Cellular thermal shift assay (CETSA).

To achieve total cellular lysis, the suspension underwent three sequential freeze–thaw repetitions using liquid nitrogen, with subsequent debris removal via a 15-min centrifugation cycle at 4 °C and 12,000 g. The resulting clarified supernatants were partitioned into eight uniform aliquots and exposed to either DMSO or 50 μM ATT-I. Each fractional sample then received a 5-min thermal challenge across a graduated temperature spectrum of 42 °C–70 °C (4 °C increments) to facilitate target protein quantification through western blot analysis. AAV interference.

To evaluate the contribution of CA9 to the medicinal efficacy of ATT-I, an adeno-associated virus 9 (AAV9) carrying a mouse CA9-specific short hairpin RNA (shRNA) was constructed and used for *in vivo* gene silencing. The shRNA sequence was designed against mouse CA9 mRNA. The target sequence of shCA9 was 5′-GCT​GTC​CCA​TTT​GGA​AGA​AAT-3′, and the corresponding hairpin sequence was 5′-GCT​GTC​CCA​TTT​GGA​AGA​AAT-TTC​AAG​AGA-ATT​TCT​TCC​AAA​TGG​GAC​A GC-TTTTTT-3′, consisting of the sense strand, loop sequence, antisense strand, and RNA polymerase III termination signal. The recombinant vector was packaged into AAV9 capsids to generate AAV9-U6-shCA9. AAV9 carrying a non-targeting scrambled shRNA sequence was used as the negative control vector (AAV9-U6-shNC). For *in vivo* administration, mice received AAV9-U6-shCA9 or AAV9-U6-shNC via intrarectal injection through the tail vein at a dose of 10^11^ vg/mouse in a volume of 100 μl before DSS treatment.

### 2.16 Statistical analysis

Statistical significance for all comparative analyses was defined by a *p < 0.05*. Using GraphPad Prism 8.0 for comprehensive data management, inter-group variances were determined through one-way ANOVA. Numerical findings are expressed as mean ± SD, reflecting the standardized approach to the quantitative evaluation of experimental results.

## 3 Results

### 3.1 Atractylenolide l (ATT-I) relieved general pathological symptoms of DSS-induced colitis in mice

To evaluate the anti-colitis activity of ATT-I, we established a mouse model of acute colitis by administering 3% DSS. Except for the control group, all cohorts exhibited weight reduction and increased disease activity index (DAI) from day 1–7. However, during the subsequent 3 days (days 8–10), the administration of SASP or ATT-I (20 and 40 mg/kg) substantially mitigated these pathological changes, with the 40 mg/kg dose demonstrating superior efficacy (Figures 1A,B). The colon length reduction observed in DSS-challenged mice was significantly reversed by these treatments (*p < 0.05* or *p < 0.01;* Figure 1C). Furthermore, compared with the DSS-only group, mice receiving ATT-I (20 or 40 mg/kg) or SASP exhibited a marked reduction in both splenic weight and the corresponding spleen index (*p < 0.01*). This reversal of splenomegaly provides additional evidence for the robust anti-inflammatory properties of ATT-I during DSS-induced colonic injury (Figure 1D). Histopathological analysis indicated that DSS induced severe structural damage, including mucosal erosion, loss of goblet cells, and extensive leucocyte infiltration (yellow arrow indicated). Notably, ATT-I (20 or 40 mg/kg) and SASP effectively preserved the mucosal integrity and curtailed inflammatory recruitment (Figure 1E). AB-PAS staining results revealed that DSS caused a massive loss of goblet cells in colonic tissues, as evidenced by markedly diminished blue-stained areas. ATT-I and SASP administration restored the number of AB-PAS-positive goblet cells (Figure 1F). These observations suggest that ATT-I alleviates DSS-triggered colonic inflammation and supports mucosal recovery.

**FIGURE 1:**
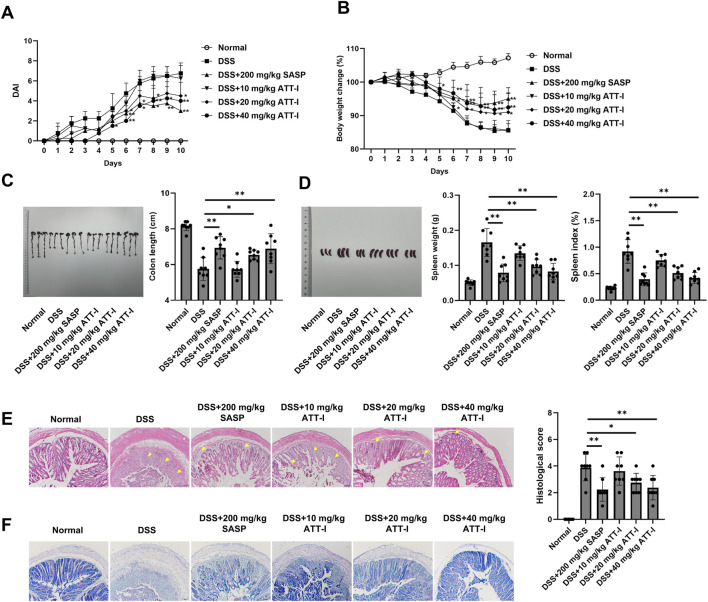
Atractylenolide l (ATT-I) relieved DSS-induced colitis in mice. **(A)** DAI score; **(B)** Body weight change; **(C)** Representative images of colon length and colon length analysis in diverse groups; **(D)** Representative images of spleen and spleen weight and index analysis in diverse groups. **(E)** Representative histological images of H&E staining and histological scores in diverse groups (scale bar = 100 μm). **(F)** Representative images of AB-PAS staining in diverse groups (scale bar = 100 μm). These data are expressed as mean ± SD, *n* = 8. * *P* < 0.05, ** *P* < 0.01.

### 3.2 ATT-I inhibited overproduction of colonic pro-inflammatory cytokines

We utilized real-time qPCR to evaluate the transcript profiles of various pro-inflammatory mediators within the colon, aiming to clarify the regulatory role of ATT-I in systemic and mucosal inflammation. As illustrated in Figure 2A, the mRNA levels of TNF-α, IFN-γ, IL-1β, IL-18, IL-6, and IL-23 were markedly suppressed in mice receiving 20 or 40 mg/kg of ATT-I when compared to the DSS-exposed group (*p < 0.05* or *p < 0.01*). This inhibitory effect was further validated at the protein level via ELISA, which demonstrated a parallel decline in cytokine concentrations following ATT-I intervention (Figure 2B). Notably, the positive control, SASP, yielded similar repressive effects across both mRNA and protein expressions. Consistent with the qPCR and ELISA result, western blot results showed that ATT-I treatment (40 mg/kg) significantly reduced the protein expression levels of TNF-α and IL-1β (Figure 2C). The therapeutic efficacy of ATT-I against colitis is largely attributable to the downregulation of pro-inflammatory cytokine production, with the 40 mg/kg dosage showing maximal potency.

**FIGURE 2:**
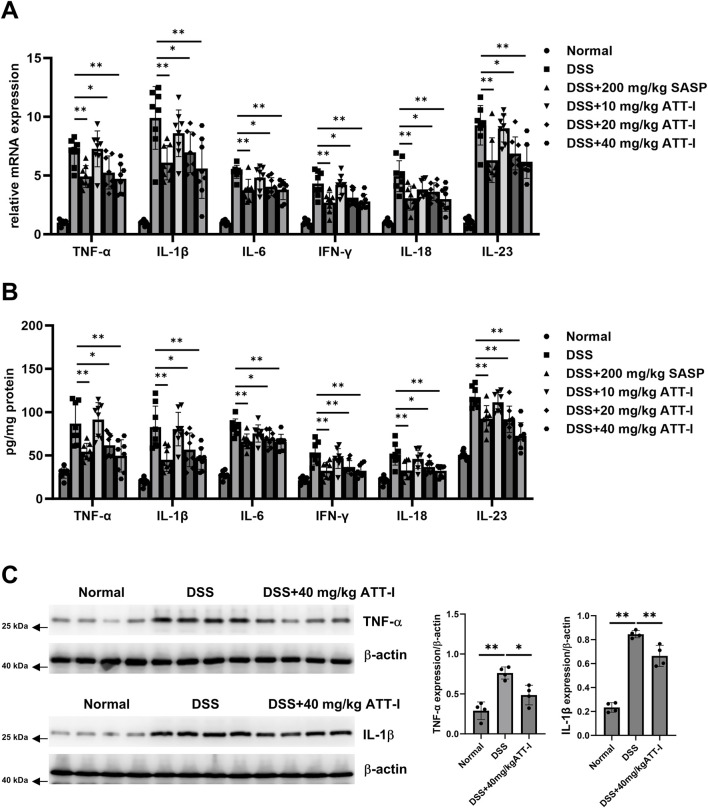
ATT-I inhibits inflammatory cytokine production in colon tissue induced by DSS. **(A)** Real-time qPCR analysis of genes, relative expression of TNF-α, IFN-γ, IL-1β, IL-18, IL-6, IL-23 (*n* = 8); **(B)** ELISA analysis of TNF-α, IFN-γ, IL-1β, IL-18, IL-6, IL-23 levels in colon tissue (*n* = 8). **(C)** Western blot analysis of TNF-α and IL-1β levels in colon tissue (*n* = 4). Using β-actin as an internal control, band quantification was performed using ImageJ software (version 1.8.0). These data are expressed as mean ± SD. * *P* < 0.05, ** *P* < 0.01.

### 3.3 ATT-I preserved intestinal barrier integrity and reduced epithelium cell death

Intercellular junctional stability relies fundamentally on specific integral membrane proteins, notably Occludin and Claudin-1 (Li et al., 2024). Protein quantification via Western blot revealed that the administration of ATT-I at 40 mg/kg significantly upregulated the expression of Occludin and Claudin-1 compared to DSS-challenged subjects (*p < 0.01*) (Figure 3A). Histological assessment via TUNEL staining revealed a substantial attenuation of cell death within the colonic architecture following administration of SASP or ATT-I. These interventions effectively suppressed the population of apoptotic nuclei, thereby confirming the anti-apoptotic properties inherent to ATT-I treatment (Figure 3B). Furthermore, the restoration of intestinal permeability was confirmed by the FITC-dextran assay, where serum fluorescence intensity was markedly lower in mice receiving 20 or 40 mg/kg ATT-I or SASP relative to the DSS group (*p < 0.05* or *p < 0.01;* Figure 3C). Consistent with these observations, serum diamine oxidase (DAO) activity, an established indicator of intestinal mechanical barrier health, exhibited a marked decline in both the SASP and ATT-I groups (*p < 0.05* or *p < 0.01;* Figure 3D). In summary, the observed results indicate that ATT-I administration effectively mitigates mucosal damage while simultaneously reinforcing the structural stability of the enteric barrier in subjects with induced colitis.

**FIGURE 3:**
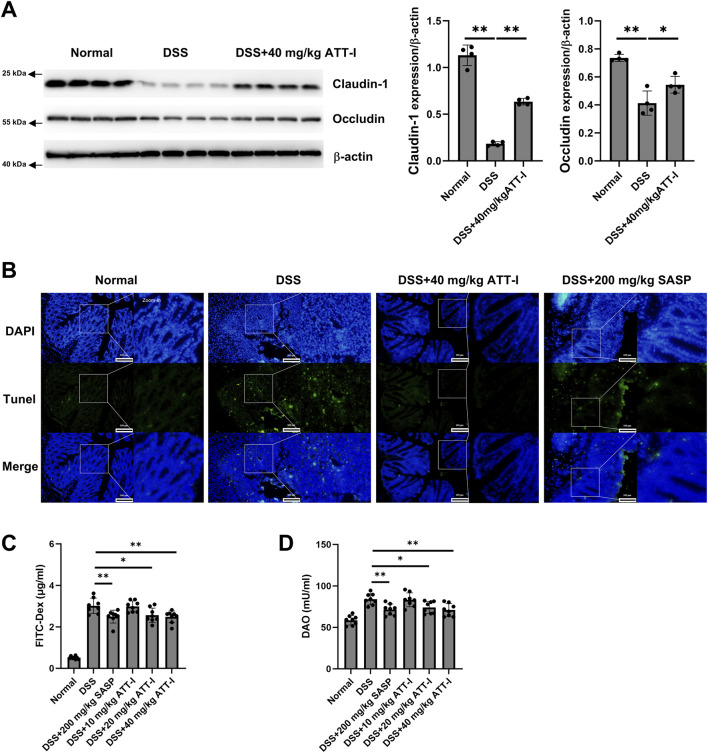
ATT-I relieved intestinal epithelium cells damage in DSS-induced colitis mice. **(A)** Effects on the expression levels of Claudin-1 and Occludin in diverse groups (*n* = 4). Using β-actin as an internal control, band quantification was performed using ImageJ software (version 1.8.0); **(B)** TUNEL staining results (scale bar = 100 μm); **(C)** Intestinal permeability to 4 kDa FITC-dextran with and without ATT-I treatment was evaluated based on optical density (*n* = 8); **(D)** Diamine oxidase (DAO) activity detection (U/g, *n* = 8). Data are presented as the mean ± SD. * *P* < 0.05, ** *P* < 0.01.

### 3.4 ATT-I exerted an inhibitory effect on ferroptosis in colon tissue induced by DSS

In order to clarify the mechanical basis of the protection conferred by ATT-I, its impact on iron homeostasis, oxidative stress, and ferroptosis-induced tissue damage was assessed. Compared to the DSS-challenged mice, ATT-I intervention (especially at 40 mg/kg) significantly lowered the concentrations of Fe^2+^ and malondialdehyde (MDA) while concurrently bolstering glutathione (GSH) levels and superoxide dismutase (SOD) activity (*p* < 0.05 or *p* < 0.01; Figure 4A). These alterations imply that ATT-I curtails Fe^2+^ sequestration and lipid peroxidation, potentially by modulating iron transport or sequestration pathways.

**FIGURE 4:**
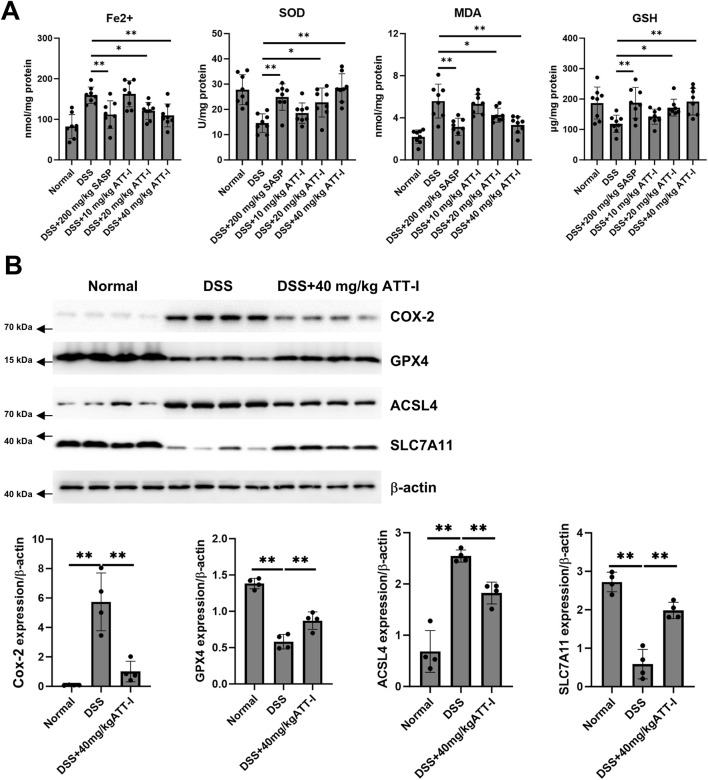
ATT-I exerted an inhibitory effect on ferroptosis in DSS-induced colitis mice. **(A)** Detection of Fe^2+^, MDA, GSH, and SOD levels using assay kits (*n* = 8); **(B)** The expression levels of ferroptosis-related marker proteins in diverse groups (*n* = 4). Using β-actin as an internal control, band quantification was performed using ImageJ software. Data are presented as the mean ± SD. * *P* < 0.05, ** *P* < 0.01.

In the context of ferroptosis, COX-2 activation is known to exacerbate inflammation and oxidative damage, thereby driving iron overload (Stockwell et al., 2017). Conversely, GPX4, ACSL4, and SLC7A11 serve as pivotal modulators in the detoxification of lipid peroxides (Yang et al., 2014). As illustrated in Figure 4B, the 40 mg/kg ATT-I treatment group exhibited a marked downregulation of COX-2 and ACSL4, alongside a robust upregulation of GPX4 and SLC7A11 (*p* < 0.01). The immunofluorescence co-staining experiments on colonic tissue sections showed that, in the DSS groups, the fluorescence intensities of GPX4 (ferroptosis marker, red) within EpCAM-positive epithelial cells (intestinal epithelial cell marker, green) were significantly reduced, indicating that intestinal epithelial cells underwent ferroptosis. After ATT-I treatment, the expression levels of GPX4 in EpCAM-positive regions were markedly restored, demonstrating that ATT-I effectively protects intestinal epithelial cells from ferroptotic damage (Figure 5). In summary, these results highlight the clinical promise of ATT-I as a therapeutic intervention aimed at suppressing ferroptosis and its linked oxidative damage.

**FIGURE 5:**
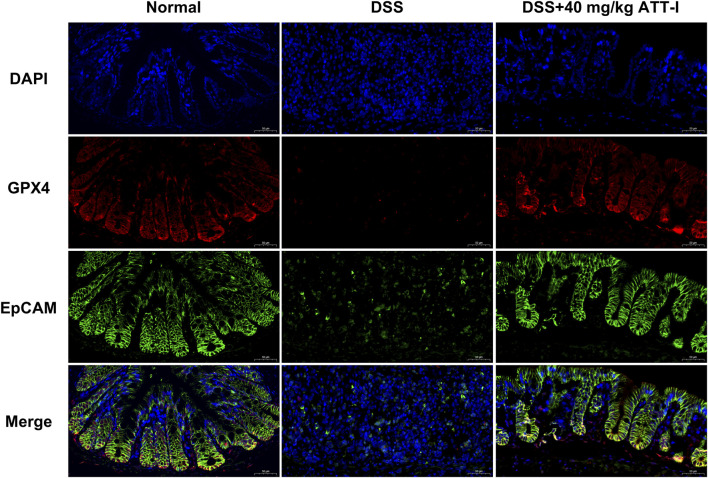
ATT-I restored GPX4 expression in intestinal epithelial cells during DSS-induced colitis. Representative confocal laser scanning microscopy immunofluorescence images of colonic tissue stained for EpCAM (intestinal epithelial cell marker, green), GPX4 (ferroptosis marker, red) and DAPI (nuclei, blue). Scale bar = 50 µm.

### 3.5 Ferroptosis inducer Erastin abrogated the protective effects of ATT-I against DSS-induced colitis

To further validate that the protective effects of ATT-I are mediated through ferroptosis inhibition, the ferroptosis inducer Erastin was co-administered with ATT-I in DSS-induced colitic mice. As shown in Figures 6A,B, ATT-I treatment significantly decreased DAI score and attenuated body weight loss compared with the DSS group, whereas co-treatment with Erastin effectively reversed these beneficial effects. Similarly, ATT-I-mediated restoration of colon length and attenuation of splenomegaly were largely abrogated by Erastin co-administration (Figures 6C,D). Histological evaluation revealed that ATT-I markedly ameliorated mucosal damage and reduced histological injury scores, while Erastin co-treatment reversed these protective effects and exacerbated colonic tissue injury (Figure 6E). AB-PAS staining further demonstrated that ATT-I substantially restored the DSS-induced loss of goblet cells, an effect that was diminished upon Erastin co-treatment (Figure 6F). Consistently, Western blot analysis showed that ATT-I upregulated the expression of the anti-ferroptotic markers GPX4 and SLC7A11, whereas Erastin co-administration abrogated this upregulation (Figure 6G). Collectively, these results confirm that ATT-I exerts its protective effects against DSS-induced colitis through inhibition of ferroptosis, as the ferroptosis activator Erastin effectively reverses all the beneficial phenotypes conferred by ATT-I. CA9 is identified as a direct molecular target of ATT-I to regulate ferroptosis.

**FIGURE 6:**
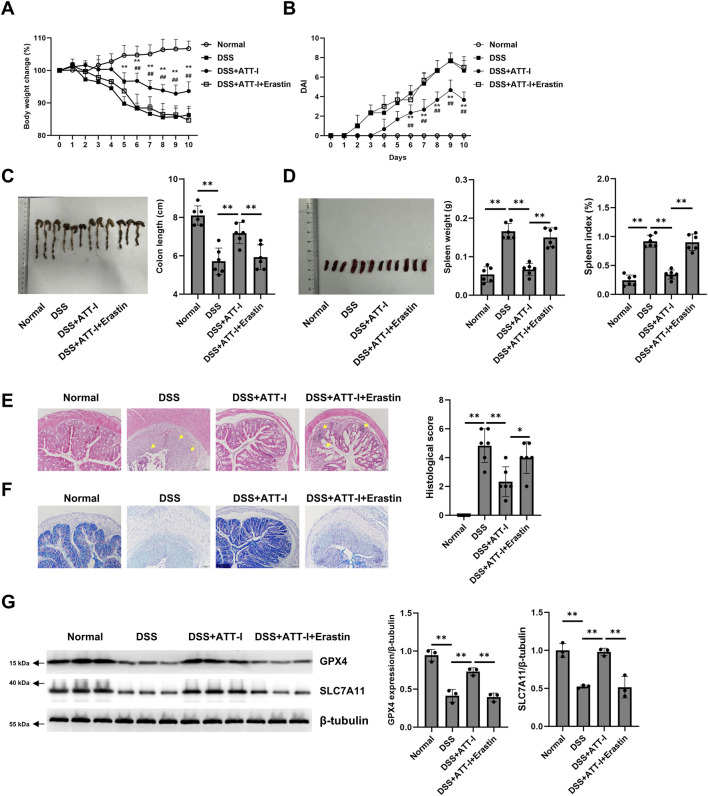
Ferroptosis inducer Erastin abrogates the protective effects of ATT-I against DSS-induced colitis. **(A)** DAI score (n = 6); **(B)** Body weight change (n = 6); **(C)** Representative images of colon length and colon length analysis in diverse groups (n = 6); **(D)** Representative images of spleen and spleen weight and index analysis in diverse groups (n = 6); **(E)** Representative histological images of H&E staining and histological scores in diverse groups (scale bar = 100 μm, n = 6); **(F)** Representative images of AB-PAS staining in diverse groups (scale bar = 100 μm, n = 6); **(G)** The expression levels of ferroptosis-related marker proteins in diverse groups (*n* = 3). Using β-tubulin as an internal control, band quantification was performed using ImageJ software These data are expressed as mean ± SD. * *P* < 0.05, ** *P* < 0.01.

The underlying regulatory influence of ATT-I on ferroptosis was next scrutinized to identify the specific signaling axis involved. Figure 7A showed the structure of ATT-I. By using Swiss Target Prediction database, we collected 27 the potential targets of ATT-I (Figure 7B). Using Genecards database, we collected 10,749 targets of IBD, 1866 targets of ferroptosis. VENN analysis showed that ATT-I may regulate ferroptosis in IBD through 7 potential targets (Figure 7C). These candidates including androgen receptor (AR), exportin-1 (XPO1), tyrosine-protein kinase receptor FLT3 (FLT3), myeloperoxidase (MPO), DNA-dependent protein kinase (PRKDC), thymidylate synthase (TYMS), were docked with ATT-I utilizing the AutoDock suite. Results highlighted CA9 as a primary candidate for ATT-I targeting, characterized by a potent binding energy of −8.131 kcal/mol (Figure 7D). CETSA was further performed to verify the interaction between ATT-I and CA9. As shown in Figure 7E, compared with the DMSO group, ATT-I treatment obviously elevated the thermal stability of CA9 protein under gradient temperatures ranging from 42 °C to 70 °C, which experimentally confirmed the direct binding of ATT-I to CA9 protein.

**FIGURE 7:**
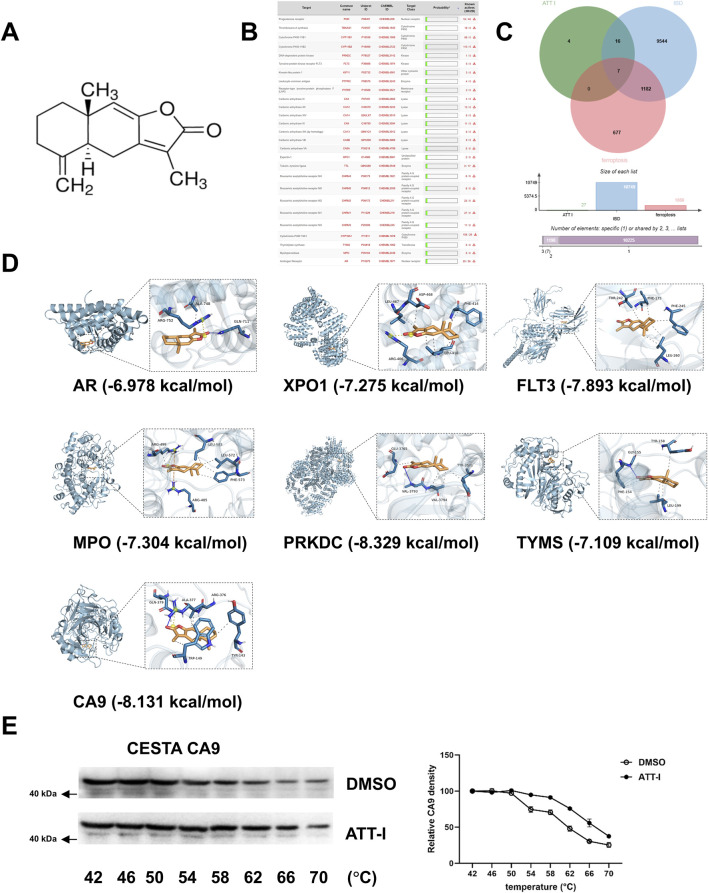
ATT-I Interacts with CA9 to Regulate Ferroptosis. **(A)** the chemical structure of ATT-I; **(B)** The 27 potential targets of ATT-I predicted by SwissTargetPrediction; **(C)** Venn analysis of the targets of ATT-I, IBD and ferroptosis; **(D)** Molecular docking of ATT-I with 7 potential targets; **(E)** The CESTA was performed using Caco-2 cell lysates in the presence of DMSO or ATT-I (50 μM). The stability of CA9 protein were determined by western blot and quantified by ImageJ software. The experiments were repeated at least three times. These data are expressed as mean ± SD.

### 3.6 Silencing CA9 abolished the protective effect of ATT-I in DSS-induced colitic mice

Using adenovirus-associated virus 9 (AAV9) to silence the CA9 gene, we investigated its critical role in the context of ATT-I-mediated suppression of ferroptosis and colitis. The results showed that, during the first 7 days, all groups of mice exhibited weight loss and increased DAI scores. Between days 8 and 10, the administration of 40 mg/kg ATT-I significantly mitigated DSS-induced weight reduction and lowered DAI values compared to the untreated model mice. However, in the DSS+40 mg/kg ATT-I + AAV-shCA9 group, the weight change was similar to the group exposed solely to DSS, and the absence of any substantial decrease in DAI scores suggests that CA9 silencing effectively neutralized the therapeutic benefits of ATT-I (Figures 8A,B).

**FIGURE 8:**
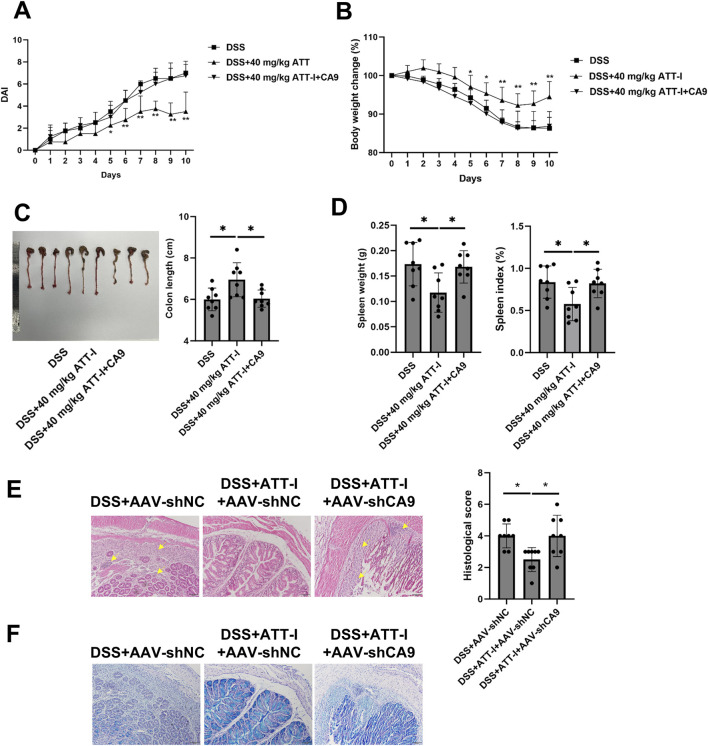
Silencing CA9 abolished the protective effect of ATT-I in DSS-induced colitic mice. **(A)** DAI score; **(B)** Body weight change; **(C)** Representative images of colon length and colon length analysis in diverse groups; **(D)** Spleen weight and index analysis in diverse groups; **(E)** Representative histological images of H&E staining and histological scores in diverse groups (scale bar = 100 μm). **(F)** Representative images of AB-PAS staining in diverse groups (scale bar = 100 μm). These data are expressed as mean ± SD, *n* = 8. * *P* < 0.05, ** *P* < 0.01.

Furthermore, as illustrated in Figures 8C,D, intervention with 40 mg/kg ATT-I significantly counteracted DSS-induced colon shortening and splenomegaly (*p* < 0.05). Notably, these therapeutic benefits were largely abrogated following the silencing of CA9. The DSS+40 mg/kg ATT-I + shCA9 group showed no significant recovery in colonic length or splenic parameters, implying that CA9 is indispensable for the protective efficacy of ATT-I. Histopathological assessment via H&E staining further corroborated these findings (Figure 8E). While ATT-I treatment effectively mitigated the severe mucosal disruption and inflammatory infiltration triggered by DSS, CA9 knockdown neutralized these reparative effects, resulting in histological damage comparable to the DSS-only group. AB-PAS staining demonstrated that ATT-I administration significantly attenuated goblet cell depletion in colonic mucosa. Conversely, knockdown of CA9 abrogated this protective effect, resulting in a marked loss of goblet cells comparable to the DSS + AAV-shNC group (Figure 8F). Collectively, these data suggest that the ameliorative impact of ATT-I on experimental colitis is mediated, at least in part, through a CA9-dependent mechanism.

### 3.7 Silencing CA9 abolished the inhibitory effect of ATT-I on inflammation and ferroptosis

Gene knockdown was used to silence carbonic anhydrase IX (CA9) and assess its requirement for the pharmacological efficacy of Atractylenolide I (ATT-I). Consistent with our initial findings, qPCR analysis showed that 40 mg/kg of ATT-I markedly lowered the mRNA abundance of various inflammatory cytokines (TNF-α, IFN-γ, IL-1β, IL-18, IL-6, and IL-23) relative to the DSS model (*p < 0.05;* Figure 9A). Remarkably, this inhibitory influence on transcript levels was effectively neutralized upon CA9 depletion, with cytokine signatures returning to levels seen in the DSS-only cohort. Parallel ELISA data confirmed that the drug-mediated reduction in protein secretion was abolished after CA9 silencing (*p < 0.05;* Figure 9B), establishing CA9 as a critical mediator of the anti-inflammatory properties of ATT-I.

**FIGURE 9:**
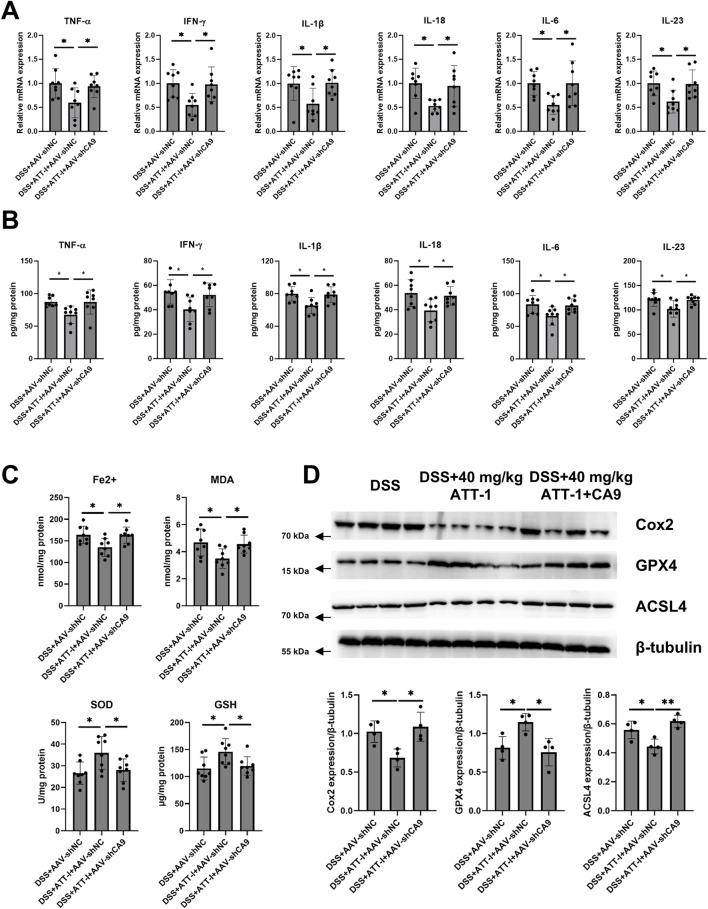
Silencing CA9 abolished the inhibitory effect of ATT-I on inflammation and ferroptosis. **(A)** Real-time qPCR analysis of genes, relative expression of TNF-α, IFN-γ, IL-1β, IL-18, IL-6, IL-23 (*n* = 8); **(B)** Contents of TNF-α, IFN-γ, IL-1β, IL-18, IL-6, IL-23 in colon tissue (*n* = 8); **(C)** Detection of Fe^2+^, MDA, GSH, and SOD levels using assay kits (*n* = 8); **(D)** The expression levels of ferroptosis-related marker proteins in diverse groups (*n* = 4). Using β-tubulin as an internal control, band quantification was performed using ImageJ software (version 1.8.0). These data are expressed as mean ± SD. *P < 0.05.

Furthermore, the influence of CA9 on iron homeostasis and redox balance was assessed. As illustrated in Figure 9C, while ATT-I (40 mg/kg) effectively curtailed Fe^2+^ and MDA accumulation while bolstering GSH and SOD levels, these antioxidant benefits were markedly attenuated in the CA9-silenced cohort. Additional Western blot results indicated that the regulatory impact of ATT-I on ferroptosis-related markers, specifically the downregulation of ACSL4 and COX-2 along with GPX4 elevation, was markedly mitigated after CA9 knockdown (*p* < 0.05, Figure 9D). Collectively, the biological data support the conclusion that CA9 serves as a requisite mediator for the pharmacological efficacy of ATT-I against colitis-associated ferroptosis and inflammatory responses.

### 3.8 Silencing CA9 abolished the inhibitory effect of ATT-I on ferroptosis in Caco-2 cells

To confirm CA9 is required for ATT-I to suppress Erastin-triggered ferroptosis, Caco-2 cells were transfected with negative control siRNA (si-NC) or CA9-targeted siRNA (si-CA9), followed by co-treatment with 20 μM Erastin and 50 μM ATT-I for 24 h. First, the CCK-8 assay was performed to test the cytotoxicity of ATT-I. The results showed that ATT-I treatment ranging from 6.25 to 100 μM for 24 h didn’t induce the reduction of cell viability, whereas ATT-I at 200 μM significantly inhibited the cell viability (Figure 10A). Figure 10B showed that co-administration of gradient concentrations of ATT-I (12.5, 25 and 50 μM) dose-dependently rescued Erastin-suppressed cell viability, suggesting that low-dose ATT-I could effectively alleviate Erastin-mediated ferroptotic damage *in vitro*. Western blot results revealed that, in si-NC cells, Erastin robustly downregulated the expression of tight junction proteins Claudin-1 and Occludin, as well as anti-ferroptotic markers GPX4 and SLC7A11 and these protein deficits were largely restored upon ATT-I co-treatment. In contrast, silencing CA9 abrogated ATT-I-mediated the upregulation of these proteins, indicating that CA9 is an indispensable mediator for ATT-I to restrain Erastin-induced ferroptosis in Caco-2 cells (Figure 10C).

**FIGURE 10:**
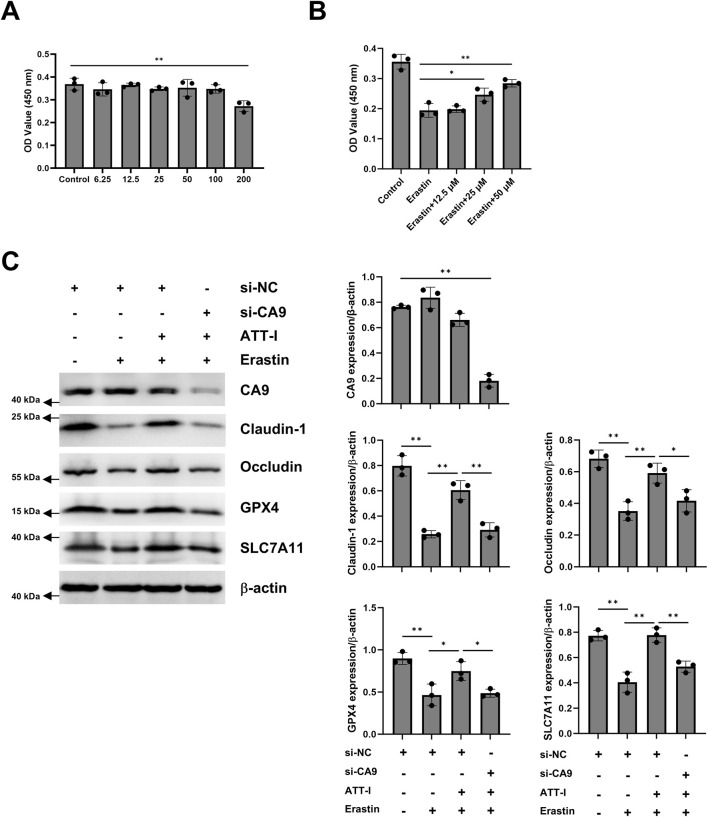
Silencing CA9 abolished the inhibitory effect of ATT-I on ferroptosis in Caco-2 cells. **(A)** Caco-2 cells were treated with different concentrations of ATT-I (6.25–200 μM) for 24 h, the cell viability was determined by the CCK-8 assay; **(B)** Caco-2 cells were treated with Erastin (20 μM) or ATT-I (12.5–50 μM) for 24 h. The cell viability was determined by the CCK-8 assay. **(C)** Caco-2 cells were transfected with si-NC or si-CA9 plasmids and then treated with Erastin (20 μM) or ATT-I (50 μM) for 24 h. Effects on the expression levels of Claudin-1, Occludin, GPX4 and SLC7A11 in diverse groups (n = 3). Using β-actin as an internal control, band quantification was performed using ImageJ software (version 1.8.0). These data are expressed as mean ± SD. *P < 0.05, ** *P* < 0.01.

## 4 Discussion

Ulcerative colitis (UC) and Crohn’s disease (CD) represent the two predominant clinical classifications of inflammatory bowel disease (IBD). This pathological state is defined as a persistent, relapsing-remitting inflammation that localized within the digestive system (Bruner et al., 2023). Due to the chronic nature of IBD, it remains challenging to achieve effective treatment outcomes, with issues such as limited efficacy, significant side effects, and drug resistance (Ribaldone et al., 2025). Natural products often exhibit good biological activity, capable of modulating various physiological and pathological processes in the body through multi-target effects. To address the clinical challenges of IBD, this investigation evaluates the efficacy of ATT-I, a naturally derived substance, in mitigating pathological symptoms. Such organic compounds have demonstrated considerable medicinal promise through their capacity to regulate immunological activity while providing robust anti-inflammatory and antioxidant protection. The study demonstrates that ATT-I can directly target CA9, exerting anti-inflammatory and iron-regulatory effects to alleviate IBD.

During IBD, inflammatory cells are activated and secrete a variety of pro-inflammatory cytokines, which not only enhance the local inflammatory response but also cause damage to intestinal epithelial cells, cell death, and disruption of the intestinal barrier function (Ma et al., 2025). Previous studies have demonstrated that ATT-I exerts therapeutic effects on a range of diseases through its anti-inflammatory activity. It was reported that ATT-I inhibited the levels of pro-inflammatory factors, and ameliorate acute liver injury in mice (Du et al., 2022; Zhang et al., 2025). ATT-I also alleviated indomethacin-induced gastric mucosal lesions through the inhibition of the NLRP3 inflammasome signaling pathway (Yuan et al., 2024). Consistently, this study elucidates that ATT-I, particularly at moderate-to-high dosages, markedly abrogates the synthesis of major pro-inflammatory markers, including TNF-α, IL-18, IFN-γ, and IL-1β. Given that these mediators serve as the primary drivers in orchestrating the pathological inflammatory cascade of IBD, their suppression highlights the remedial potential of ATT-I. Additionally, histological improvements were observed, as well as enhanced integrity of the intestinal epithelial cells, suggesting that ATT-I provides protective effects on the intestinal barrier.

In the context of enteric inflammatory processes, ferroptosis represents a vital mechanism of programmed cellular demise. This pathway is fundamentally defined by the accumulation of lipid-based oxidative damage alongside the dysregulation of systemic or cellular iron homeostasis. In the context of DSS-mediated UC, this iron-driven process has been associated with the modulation of the Keap1/Nrf2 pathway (Chen D. et al., 2025). The role of ferroptosis in compromising epithelial integrity was further elucidated through the identification of NF-κB p65 as a pivotal inhibitory factor (Xu et al., 2020). Moreover, the attenuation of ferroptotic damage in colitis via GPX4 induction has been documented with agents like Butyrate, Hesperetin (Chen et al., 2024; Wang et al., 2024). Taken together, existing evidence supports a strong association between ferroptosis and IBD pathogenesis. To date, however, the role of ATT-I in ferroptosis remains completely unknown. One study revealed that a traditional Chinese medicine Huoxue Rongluo formula restored GPX4 expression and improved cerebral ischemia-reperfusion injury through inhibition of ferroptosis, while atractylenolide III is one of the active constituents of this formula (PMID:40744419). In this study, we identify ATT-I as a dual-acting regulator that enhances GPX4 while downregulating COX-2, culminating in ferroptosis resistance for the first time. COX-2 is a fundamental driver for the orchestration of inflammatory responses and oxidative stress. The concurrent modulation of these pathways suggests that ATT-I exerts both anti-inflammatory effects and protection against ferroptotic epithelial injury, processes implicated in IBD. The results indicate that the attenuation of DSS-induced colitis by ATT-I is mediated through regulation of ferroptosis.

In this study, molecular docking evidence identifies CA9 as an essential target of ATT-I, a finding supported by the fact that silencing CA9 expression eliminated the protective influence of ATT-I within the context of DSS-driven murine colitides. Prior work validated the barrier-protective and anti-inflammatory capacity of ATT-I in UC via cytoskeletal pathway (Gao et al., 2025), while our study further uncovered a previously unreported CA9-dependent anti-ferroptosis branch, expanding the multi-target pharmacological spectrum of ATT-I. CA9 belongs to the carbonic anhydrase family, which is fundamentally characterized by its ability to facilitate the reversible hydration of carbon dioxide (Giovannuzzi and Supuran, 2025). The result indicates that CA9 is the primary target through which ATT-I modulates ferroptosis to alleviate IBD. It has been reported that ATT-I restore intestinal barrier function by targeting the S100A9/AMPK/mTOR signaling pathway (Chen C. et al., 2025). Our finding adds a new layer of understanding to the mechanism of action of ATT-I. CA9 is an enzyme involved in maintaining acid-base balance. The interaction between ATT-I and CA9 may help modulate the acid-base balance in the intestinal mucosa, thereby producing anti-inflammatory and ferroptosis-inhibitory effects. This mechanistic finding provides a solid foundation for further research aimed at establishing the functional requirement of CA9 for pharmacological intervention in colitis.

Although these results provide a preliminary basis for the medicinal application of ATT-I in treating intestinal pathologies, further investigation is necessary. A rigorous exploration of the intricate signaling pathways involved, coupled with extensive verification across varied pathological frameworks, remains essential to fully characterize its pharmacological profile. Despite the encouraging outcomes observed in this murine model, the translational relevance of ATT-I necessitates rigorous clinical investigation. Furthermore, establishing the long-term safety profile and therapeutic durability of ATT-I in human cohorts is a prerequisite for its potential integration into clinical practice.

In summary, this study identifies that ATT-I is an effective candidate for IBD treatment, acting through the modulation of ferroptosis and targeting CA9. In light of the escalating global burden of IBD and the constraints of existing pharmacotherapies, Atractylenolide I (ATT-I) represents a promising and innovative intervention for disease management. Definitive ascertainment of the medicinal effectiveness of ATT-I for those afflicted with IBD requires the implementation of rigorous clinical trials and further forward-looking research. Meticulously structured human studies are essential to substantiate the healing potential observed in preliminary models and ensure translatable results.

## Data Availability

The raw data supporting the conclusions of this article will be made available by the authors, without undue reservation.
